# Addressing supply side constraints for digital work creation: A Concurrent Embedded Mixed Method Study in Kenya

**DOI:** 10.12688/openresafrica.16307.2

**Published:** 2026-06-12

**Authors:** Kellen Kiambati, Ehud Gachugu, Anne Kariuki

**Affiliations:** 1 Karatina University, HRD, Karatina, Nyeri Country, 1957 - 10101, Kenya; 2 Kenyatta University, Nairobi, 43844-00100, Kenya; 3 Karatina University, Nyeri, 1957-10101, Kenya

**Keywords:** Digital Work Opportunities; Demographic Factors; Training and Mentorship; Awareness Creation, Accessibility

## Abstract

**Background:**

This paper addresses a pathway for addressing high unemployment among educated and skilled youth. The supply-side constraints limiting participation in dignified digital work remain underexplored. The aim was to examine how access to infrastructure, training and mentorship, and awareness creation influence engagement in digital work, and how demographic factors moderate these relationships.

**Methods:**

A mixed-methods concurrent design was used, combining quantitative data from a cross-sectional survey with qualitative insights gathered through focus group discussions (FGDs) and key informant interviews (KIIs).

**Results:**

Findings indicate variations in awareness of digital work, training and mentorship access, infrastructure access, and engagement across all demographic categories. Gender showed a negative correlation with infrastructure access and awareness but did not significantly relate to training and mentorship or engagement. Region, setting, age, education, and occupation all significantly correlated with digital work engagement and access levels. Moderation analysis revealed that demographic factors except gender and occupation strengthened the relationship between infrastructure access and engagement in digital work. Only regional diversity enhanced the relationship between training/mentorship and engagement, indicating generally equitable access across other demographic groups. Awareness levels and engagement were strengthened by region, setting, age, occupation, and education, but not by gender, demonstrating balanced gender influence. Overall, training and mentorship programs exhibited the most balanced implementation across demographics, while gender mainstreaming efforts appear to be yielding positive outcomes.

**Conclusions:**

The study found that work initiatives must integrate demographic considerations into the design of infrastructure, training, mentorship, and awareness programs. It make contribution by providing new evidence to guide policymakers in developing targeted interventions that expand dignified digital work opportunities as a strategy for reducing unemployment.

## Introduction

Today the unemployment rates stand at 5.1 per cent characterized by increasing inequalities, plateaued productivity rates, decreasing disposable income and poor living standards ( International Labour Organization (ILO), 2023). The estimation of a persistent working poverty has shown the growth is predicted to be on average one million and moderate poverty by eight million ( OECD 2020). In Kenya, unemployment rate stands at 5.30 percent and the Government of Kenya (GoK) has implemented several interventions, such as Ajira and Kazi kwa Vijana to curb the challenge ( GoK, 2023). Despite government efforts, youth unemployment remains very high suggesting the current interventions and enables may be under theorized. For that reason, understanding factors that hinder or enable employment in upcoming sectors such as digital economy is critical for ensuring effective interventions.

The digital economy encompasses a broad spectrum of activities from ride-sharing and freelance work to online marketplaces and microtask platforms. It has grown significantly owing to technological advancements and changing workforce preferences ( Katz & Krueger, 2016). The digital gig economy, characterized by short-term, flexible work arrangements facilitated through online platforms, has become a defining feature of the contemporary labor market ( ILO, 2016). In terms of key trends, the expansion has occurred across various sectors and industries ( De Stefano & Aloisi, 2018) and diverse forms of gig work, including labor, professional services, and creative work ( Foley & Kerr, 2019; Nyqvist, R., Peltokorpi, A., Lavikka, R., & Ainamo, A., 2025; Cristian C. Osorio-Gómez, Rodrigo F. Herrera, Laura Tupėnaitė & Eugenio Pellicer, 2025; Naresh Gupta, Indra Gunawan & Rajeev Kamineni, 2025).

Additionally, there are ongoing debates on worker classification, labor rights, and platform regulations ( Sundararajan, 2016). Digital gig economy jobs have impacts ranging from economic opportunities for individuals seeking flexible employment ( Manyika et al., 2016), creating income stability, social protection, and job security ( Wood et al., 2019) as well to increased policy discussions on labor market regulations and worker benefits ( Farber, 2015). On the African Continent, the digital gig economy has gained traction across Africa, driven by increasing mobile phone penetration and a youthful, tech-savvy workforce ( World Bank, 2019). Platforms such as Uber, Upwork, and Jumia are expanding in their presence ( ILO, 2018). This has led to opportunities for youth employment and entrepreneurship (African Development Bank, 2018), digital inclusion, technology access, and skills development ( Graham & Hjorth, 2018), regulatory complexities; taxation issues; and concerns about data privacy ( Masadeh et al., 2021). Kenya stands out as a significant player in the African digital gig economy, with platforms such as Sendy and Ajira Digital offering gig work opportunities ( Mutonyi & Okiror, 2020). Factors driving growth include high Internet penetration and mobile money usage ( Gizaw & Mengistu, 2019). This impact has enhanced opportunities for youth employment and income generation (Wambugu et al., 2021), gender disparities in gig work participation and earnings ( Muchira et al., 2021) and regulatory complexities, including taxation and platform accountability ( Ikiara, 2019). The digital gig economy represents a transformative force in the global labor market, with distinct features and implications for Africa and Kenya. While it offers new opportunities for employment and entrepreneurship, it also raises complex challenges related to labor rights, income inequality, and regulation. Continued research is essential to understand the evolving dynamics of the digital economy in these contexts and inform effective policies that ensure equitable outcomes ( Hanjing Zhu, Yuxin Feng, Bon-Gang Hwang & Xiaoxuan Fan, 2025; Junyoung Park, Sunkuk Kim & Jeeyoung Lim, 2025 and Namila Chathuranga, Fayad Thajdeen & Chandana Siriwardana, 2025). Earlier studies have investigated digital work but limited research has focused on supply side constraints and therefore the importance of this study lies in providing a scientific modeming and explanation on supply side constraints for digital work creation.

## Objectives

The main objective was to exploit differences in workers’ experiences in the gig economy to estimate the effect of access to infrastructure, training and mentorship, and awareness creation on access to dignified digital work, with the moderating effect of demographics. Different regions in Kenya adopted different strategies and some became vibrant, whereas other areas were not vibrant. The key focus is to examine the fundamental causes of the large differences in income per sector per region in Kenya.

## Literature review

Previous studies such as one carried out by ( ILO, 2023) indicates Kenya experienced strong economic performance in the 1960s and 1970s, with growth rates of over 7 per cent, largely driven by the agricultural sector, which expanded at an average rate of 4.7 per cent. The trend, however, began to weaken in the mid-1980s with the GDP growth slowing to an average of 5 per cent between 1986 and 1990 accompanied by a deterioration in key social welfare indicators. Economic conditions further declined in the early 1990s, resulting in a severe crisis by 1993. During this period, agricultural output contracted by approximately 3.9 per cent per year, while inflation surged to nearly 100 per cent. This downturn was attributed to a combination of external shocks, including the global economic slowdown and oil price increases of the 1970s, as well as challenges arising from domestic policy mismanagement.

Further evidence paints inconsistency with the earlier vision where during the e 2008 global financial crisis had a negative effect on Kenya’s economy, leading to reduced demand for exports, lower inflows of foreign investment, and depreciation of the Kenyan shilling. The downturn also produced spillover effects, including a decline in tourism revenues and remittances from Kenyans living abroad. In response, the government introduced a range of interventions, most notably the rollout of Vision 2030, aimed at positioning Kenya as a prosperous middle-income country by 2030. Financial support was extended to banks and businesses, alongside the adoption of fiscal and monetary stimulus policies. Measures were also taken to strengthen the investment environment and attract foreign direct investment. As a result, Kenya recorded relatively stable economic growth, averaging about 5 per cent between 2010 and 2019 ( World Bank, 2023). This performance was driven by increased domestic and foreign investment, strong domestic demand, enhanced macroeconomic stability, and greater sectoral diversification, which reduced vulnerability to external shocks and improved overall economic resilience ( del Torso, Ottavio, 2019; Delronge, Cynthia, Rossana Ducato, Anne-Grace Kleczewski, Enguerrand Marique, Alain Strowel and Céline Wattecamps, 2019; Digital Future Society, 2019 and Mbego, Steve, 2022).

Kenya has recorded a notable increase in mobile subscriptions, alongside a steady rise in smartphone ownership, which currently stands at 58.3 per cent ( CAK, 2023). However, despite this widespread access to mobile devices, most users primarily rely on their phones for basic digital functions. This is largely due to limited familiarity with more advanced digital applications and services, as well as challenges related to language. Gender disparities are also evident, with approximately 43 per cent of women reporting that they require assistance to use digital services, compared to 31 per cent of men, reflecting women’s lower levels of device access, ownership, and usage. In addition, older adults (69 per cent), rural populations (73 per cent), and persons with disabilities (69 per cent) are significantly more likely to need help when using mobile phones than the general population ( Abuya, Kenn, 2023; Ayugi, Mary Evelyn, Odera Odhiambo, Ondiek Gerald Ochieng, Abeka Evans, Obange Nelson and Anyango Christine, 2013; and Bryson, Alex, Richard Freeman, Rafael Gomez and Paul William, 2019). The review of literature has identified research gaps addressed in this study.

## Theoretical framework

The study is anchored on three theories namely human capital theory, digital divide theory and labour market segmentation theory. According to human capital theory, labour market outcomes should be strongly influenced by the worker's educational level Calderón-Gómez, Casas-Mas, Urraco-Solanilla and Revilla (2020). Sweetland (1996) argues that people’s investment in education and skills have a direct implication on their productivity and economic outcomes. Shaw, Fiers, and Hargittai (2023) posits that in digital economy, differences in accessing training and mentorship interventions create varying engagement levels. Mincer (1993) in his book on studies in human capital indicates that most interventions have focused on formal employment set up. This study extends Human Capital Theory by demonstrating that in the context of Kenya’s digital work, training and mentorship programs have the most balanced implementation across demographic groups challenging the assumption that skills gaps are uniformly distributed.

The other primary theory considered in the study is the digital divide theory which seek to the digital divide theory attempts to provide explanation beyond accessibility of technology to competencies and the differences between skills and usage coupled with tangible outcomes ( van Dijk, 2017). Ragnedda (2017) argues digital divide simply extends traditional forms of inequality to include new trends that alleviate traditional inequalities and form new modalities of inequality. She argues that inequalities that exist in the digital sphere are certainly entangled with inequalities present in the social sphere. In this study we focus on disparities and tangible outcomes deviating from all other existing studies which largely pay attention to skills only and that forms the novelty of the study.

The last theory is the Labor Market Segmentation Theory where ( Peck, 1989) argues that labour markets are demarcated into primary and secondary parts and demographic parameters such as gender, region and education levels form the determination of whether individuals enter the sectors or not. Complementing the argument is Vietorisz and Harrison (1972) where they view segmented labor markets as an operation of a modern industrial market society. The study views the digital gig economy in Kenya in the same lens revealing demographic factors as parameters for segmentation.

## Study scope

This national study randomly targeted respondents aged 18 years and above on their participation in the digital work economy. The sample was meant to allow for a robust analysis based on demographics, specifically age, sex, geographical location, education level, occupation, and setting. This survey highlights the various factors that programs seeking to enhance access to digital work among youth in Kenya should consider their activities in a bid to enhance the effectiveness of their programs. The influence of various demographic factors, infrastructural access, training and mentorship, and awareness creation on accessibility to digital work were reviewed in this survey.

## Methodology

Prior to starting the study, approvals were sought from NACOSTI in Kenya Ref No: 940056 on 24 ^th^ August, 2023. A concurrent mixed approach was adopted for this study, comprising a cross sectional survey and Key Informant Interviews undertaken at the same time. A two stage cluster sampling was done in line with Kenya household survey framework ( Kenya National Bureau of Statistics, 2010). Weighting was done to take are of differentials in strata. An iterative approach was used to develop the survey questionnaire and Key Informant Interviews and it had both open and closed ended questions. A pilot study was done using fifty respondents who were not included in the final sample. For the quantitative component, a nationally representative sample of 1,917 interviews was used. This sample was used to provide information on programme indicators at the country level. In addition, a boost sample of 317 digital workers was included to enable a detailed quantitative analysis to generate insights into the digital economy of the workers. This sample of digital workers has been analysed together with the national sample to provide more insights into gig economy workers. The qualitative component consisted of in-depth interviews (IDIs) to generate in-depth insights into workers' experience in the gig economy. Ten (10) in depth interviews (IDIs) were conducted with both male and female digital workers as part of the qualitative component. The inclusion criteria was one to be active digital worker with experience undertaking digital enabled tasks. The tasks were categorized as short term, project based and long term. Written informed consent for participation in the study was obtained and participants were continuously informed of their right to pull out of the study if need be. The data collection tools indicated the same in writing.

### Operationalization of key variables

Each construct of the study was operationalized through a scale development in line with Leach Sankofa (2022) argument that developing good measurement takes iterative mixed approach. For each variable, indicators were developed. Binary parameters were converted to ratio scales using proportional measurement and comparable scale to allow for regression analysis as shown in the results section of the paper.

### Data presentation and measurement

Data were collected using qualitative and quantitative forms. The data captured in binary form were presented as 0 and 1, thus making them quantitative, while the categorical indicators with reversed ratings were reversed to ensure that all the indicators of a variable were unidirectional. Each of the four main study variables was measured using several indicators; hence, each indicator was converted into a ratio to ensure that all were using the same scale, with the final data being the ratio scale in nature. The independent variables were all measured on a ratio scale: Infrastructure Access had a ratio scale ranging from to 0-1, with a mean of 0.3136; Training & Mentorship had a ratio ranging from 0-5 to with a mean of 0.2262, while awareness level was measured as a ratio variable ranging between 0-5 with a mean of 1.4657. Notable was the fact Although the scale of training and mentorship was 1-5, the mean rating was 0.2262, revealing a very low average rating for this variable, which is surprising given the many opportunities available for digital skills generation. The dependent variable, Digital Work Engagement Level, was measured on a ratio scale ranging from to 0-3 with a mean rating of 0.2921, revealing that a large proportion of respondents had participation levels. The demographic variables were measured as categorical variables, with age (10 categories: mean 4.4459) and education level (7 categories, mean 4.8807) being ordered as categorical variables and gender (2 categories, mean 1.3276 showing higher participation of women), region (8 categories, mean 4.47), occupation (7 categories with a mean of 3.9804), and setting (3 categories with mean 1.41) as distinct categorical variables. The study variables are therefore quantitative and ideal for descriptive and inferential analyses. The variables used are listed in Table 1.

**Table 1.** Variables estimates summary descriptive.

Variable	Mean	Std. Dev.	Min	Max	Type of variable
Infrastructure Access	.3136	.241	0	1	Ratio
Training and Mentorship	.2262	.741	0	4.653	Ratio
Overall Awareness level	1.4657	1.166	0	5	Ratio
Digital Work Engagement Level	.2921	.463	0	3	Ratio
Age	4.4459	2.504	1	10	Categorical (10 Categories)
Occupation	3.9804	1.408	1	7	Categorical (7 Categories)
Education	4.8807	1.833	1	7	Categorical (7 Categories)
Gender	1.3276	.469	1	2	Categorical/Binary
Setting	1.4100	.607	1	3	Categorical (3 Categories)
Region/Location	4.4700	2.374	1	8	Categorical (8 Categories)

### Data analysis

Data were analyzed using descriptive and inferential statistics. Measures of central tendency and dispersion were included in the descriptive statistics. Inferential statistics were used to test the hypotheses and examine the relationship between the study variables. Regression analysis, correlation analysis, and goodness-of-fit tests have been extensively used. The survey used a hierarchical regression model to investigate the direct, joint, and moderating effects of demographic factors, access to infrastructure, training and mentorship, and awareness creation on the level of access to the digital economy in Kenya. The regression models showed how much of the total variance in the independent variable was due to independent and moderating variables. The software SPSS and STATA were utilized for analysis, as they can analyze complex statistical models at the same time. In addition, diagnostic tests were conducted to verify that the study met all the model assumptions to inform the survey hypothesis. Qualitative data were analyzed using content analysis techniques. The results of the data analysis are presented in tables and graphs. Additionally, the survey undertook qualitative data analysis, where thematic analysis was undertaken to identify, categorize, analyze, and interpret patterns in the qualitative study data. Each transcribed interview was first analyzed individually to arrive at an understanding of each participant. Common discourses were then identified, focusing specifically on the ways in which participants constructed the discourses under investigation.

Each transcribed interview was first analyzed individually to arrive at an understanding of each participant, thematic analysis was undertaken. Common discourses were then identified, focusing specifically on the ways in which participants constructed the discourses under investigation. The qualitative data was transformed and quantitatized for analysis. The aim was to increase the explanatory power ( Halevi Hochwald, Green, Sela, Radomyslsky, Nissanholtz-Gannot, & Hochwald, 2023; De Block & Vis, 2019).

## Study findings

Upon data analysis, the presentation and interpretation of the findings were undertaken in this section, highlighting descriptive and inferential statistics.

### Descriptive analysis

Descriptive analysis was undertaken to understand how the various survey variables presented themselves in the study and the state of the indicators as measured in the study. Descriptive statistics included frequencies, percentages, means, and standard deviations. Descriptive demographic information regarding access to digital infrastructure is presented in Table 2.

**Table 2.** Access to infrastructure along varying demographics.

	Access to Laptop/Computer		Access to smartphone		Access to Tablet/Ipad		Access to the internet	
Region	Freq.	Percent	Freq.	Percent	Freq.	Percent	Freq.	Percent
Nairobi	125	46%	233	85%	40	15%	235	86%
Central	93	29%	205	63%	24	7%	208	64%
Eastern	36	16%	132	60%	14	6%	121	55%
North Eastern	24	19%	83	66%	10	8%	78	62%
Coast	34	20%	111	64%	13	8%	109	63%
Rift Valley	88	22%	241	59%	33	8%	256	63%
Western	34	15%	121	54%	12	5%	127	56%
Nyanza	39	16%	130	54%	18	8%	132	55%
**Setting**								
Rural	197	15%	701	54%	72	6%	717	55%
Urban	222	39%	451	79%	67	12%	452	79%
Peri-urban	54	43%	104	83%	25	20%	97	78%
**Gender**								
Male	350	26%	891	67%	123	9%	916	69%
Female	123	19%	365	56%	41	6%	350	54%
**Age**								
18-24 years	78	36%	182	83%	23	11%	182	83%
25-29 years	125	43%	235	80%	34	12%	238	81%
30-34 years	111	35%	237	74%	31	10%	241	75%
35-39 years	59	21%	191	67%	32	11%	193	67%
40-44 years	42	16%	150	57%	19	7%	153	58%
45-49 years	22	13%	84	52%	11	7%	88	54%
50-54 years	16	8%	87	45%	6	3%	90	47%
55-59 years	10	15%	29	43%	2	3%	30	44%
60-64 years	4	5%	34	44%	5	6%	27	35%
65+ years	6	6%	27	26%	1	1%	24	23%
**Grand Total**	**473**	**24%**	**1256**	**63%**	**164**	**8%**	**1266**	**64%**

The study found that access to smartphones was high within the studied population, with 63% of people having access to a smartphone. Access to the Internet was observed to be higher at 64%, because anyone with a smartphone is considered to have access to the Internet. Access to laptops/computers was observed to be far lower at 24% of the studied sample, revealing low accessibility to these devices, while access to a tablet, which is the most plausible replacement, was observed to be at only 8%, revealing that very few people have access to these devices. These observations confirm that smartphones are the most accessible technological devices that have widened accessibility to the Internet for more people in the country. Laptop and computer access were significantly less accessible to only 24% of the interviewed respondents. The Nairobi and Central regions revealed significantly higher accessibility to digital infrastructure than the other regions, with the least access observed in the Western, Nyanza, and North Eastern regions. Accessibility increases significantly within the 18-35 years age-group, where 43% of age 25-29 have access, 36% at age 18-24 years, and 35% at–30-34 years old. Similarly, these age groups have higher access to smartphones and the Internet, as they have significantly higher access to tablets/ads. Higher access was observed among male respondents than among their female counterparts. Those living in peri-urban settings were observed to have higher access to laptop/computers, smartphones, Tablet/Ipad, and internet than those living in the urban setting, which was unexpected as urban regions are expected to have higher access to digital tools than peri-urban regions. As expected, those living in urban settings have higher access to digital infrastructure than those living in rural settings. The findings are consistent with the Association for Progressive Communications (2025) where they argue connectivity alone is not sufficient and inequalities in economic activities can be occasioned by infrastructure gap.

The study examined the independent variables in the lenses of demographic variables, revealing how they manifest within varying demographic settings. The results are shown in Table 3.

**Table 3.** Average level of independent variables within different demographic settings.

Demographic Characteristics	Awareness Level	Training and Mentorship	Infrastructure Access	Engagement in Digital Work
**Region**	**Average**	**Average**	**Average**	**Average**
Nairobi	2.0367	0.5046	0.4343	0.5343
Central	1.5550	0.2686	0.3282	0.3170
Eastern	1.3169	0.1165	0.2799	0.2445
North Eastern	0.9467	0.0473	0.2949	0.1616
Coast	1.2655	0.2087	0.3097	0.2254
Rift Valley	1.4588	0.2051	0.3008	0.2691
Western	1.4240	0.1850	0.2584	0.2284
Nyanza	1.2949	0.1321	0.2732	0.2402
**Grand Total**	**1.4657**	**0.2262**	**0.3136**	**0.2921**
**Setting**	**Average**	**Average**	**Average**	**Average**
Rural	0.2659	0.1163	1.2803	0.2098
Urban	0.3983	0.4205	1.7890	0.4291
Peri-Urban	0.4206	0.4773	1.9080	0.5184
**Grand Total**	**0.3136**	**0.2262**	**1.4657**	**0.2921**
**Gender**	**Average**	**Average**	**Average**	**Average**
Male	0.3312	0.2214	1.5492	0.3019
Female	0.2776	0.2361	1.2944	0.2719
**Grand Total**	**0.3136**	**0.2262**	**1.4657**	**0.2921**
**Age**	**Average**	**Average**	**Average**	**Average**
18-24 years	0.3794	0.6235	1.9555	0.5881
25-29 years	0.3974	0.5936	2.0052	0.5358
30-34 years	0.3694	0.2604	1.7010	0.4050
35-39 years	0.3267	0.1558	1.4332	0.2538
40-44 years	0.2895	0.0106	1.2770	0.1472
45-49 years	0.2542	0.0275	1.3610	0.1460
50-54 years	0.2247	0.0200	0.9600	0.0760
55-59 years	0.2437	0.0016	1.0677	0.0853
60-64 years	0.2253	0.0007	0.8655	0.0744
65+ years	0.1609	0.0031	0.5625	0.0350
**Grand Total**	**0.3136**	**0.2262**	**1.4657**	**0.2921**
**Occupation**	**Average**	**Average**	**Average**	**Average**
Still in school/Student	0.4477	0.7456	2.4410	0.6902
Employed-working in the formal sector	0.4748	0.2844	1.8424	0.4044
Employed-working in informal sector	0.3393	0.2518	1.6275	0.3650
Self employed	0.2934	0.1646	1.4098	0.2344
Unemployed but worked past 12 months	0.2677	0.3008	1.4626	0.2736
Unemployed didn’t work past 12 months	0.2266	0.1661	1.0434	0.2272
Incapacitated/unable to work	0.1486	0.0042	0.6667	0.0400
**Grand Total**	**0.3136**	**0.2262**	**1.4657**	**0.2921**
**Education Level**	**Average**	**Average**	**Average**	**Average**
None - I did not attend school	0.1027	0.0008	0.6172	0.0406
Some Primary	0.1582	0.0155	0.8583	0.0763
Completed Primary	0.1702	0.0168	0.9362	0.0859
Some Secondary	0.1996	0.0510	1.1043	0.1308
Completed Secondary	0.2819	0.0526	1.2779	0.1592
Some college/university	0.4286	0.5887	2.0788	0.5480
Completed College/University	0.4832	0.5103	2.0976	0.5726
**Grand Total**	**0.3136**	**0.2262**	**1.4657**	**0.2921**

As shown in Table 3, it was observed that Nairobi and Central regions, which have larger urban and peri-urban populations, have the highest ratings for awareness level, training and mentorship, infrastructure access, and digital work engagement, while the other regions showed lower ratings of these factors. This shows the level of participation based on region, with predominantly lower participation among rural settings. This was confirmed when looking at the ratings based on location settings, where urban and peri-urban areas showed higher average ratings for the four main variables, revealing higher participation in these two settings. Although the highest ratings for these factors were recorded in urban settings, the peri-urban setting revealed higher levels of infrastructure access (mean 1.9080), awareness (mean 0.4206), training and mentorship (mean 0.4773), and engagement in digital work (mean 0.5184). This can be linked to the fact that the Kenyan middle sector, which has wider networks, greater access to information, and greater access to digital tools, moved from urban areas and is predominantly located in peri-urban settings. Furthermore, the survey found that male respondents had higher ratings for Awareness Level, Infrastructure Access and Engagement in Digital Work, while female respondents had slightly higher ratings for Training and Mentorship, revealing that male respondents have greater access and engagement in most of the activities surrounding digital work engagement. The four factors are more accessible to young people below the age of 35, with the highest rating of all four being reported among the young respondents involved in the study. Among the elderly, very low ratings were observed, especially on access to training and mentorship, as well as engagement in digital work, revealing very low engagement in digital work within this population segment. Those still in school had the highest ratings for digital work. Those in formal and informal employment had higher relative average ratings for the employment factors, but lower ratings among the unemployed and those in self-employment were unexpected, although this might be due to the fact that the majority of those engaged in digital work consider themselves to be in formal employment or self-employed. It was also observed that the higher the education level, the higher the ratings indicated for digital work indicators, with the highest ratings observed among those with the highest educational achievement and lowest ratings among those without any education. This can be linked to the high educational requirements required for digital work engagement. The results align with a recent study by the World Bank (2026) indicating that lack of devices and reliable internet are foundational barriers to digital inclusion.

### Inferential statistics

To generate the desired statistical inference, allowing for generalization of the study sample to the studied population, the study undertook an inferential analysis of the data. Some of the inferential statistical tools utilized include correlation, principle component analysis, and hierarchical regression models. The outcomes are presented in this section.

### Factor analysis

The factor analysis undertaken in this survey utilized principal component analysis (PCA) as the statistical technique used for data reduction. The PCA assessment has good values for all variables from the KMO-MSA, with the overall value being high enough at 0.835, and Bartlett’s Test of Sphericity has an associated P value (sig in the table) of <0.001, revealing that the results of the factor analysis would be considered valid as the correlation matrix is the identity matrix in the model. It is notable that both the factor loading and component matrix extracted three factors. However, the factor loadings (or strictly speaking the component loadings for the PCA) for the PCA are larger in absolute values, as are the communalities; consequently, the total variance explained is also greater with the ability to cumulatively explain 56.4% of the total variance. The three rotated factors are just as good as the initial nine factors in explaining and reproducing the observed correlation matrix (thus confirming the value of the three independent variables). The results are shown in Table 4.

**Table 4.** Principal component analysis.

KMO and Bartlett’s Test		
Kaiser-Meyer-Olkin Measure of Sampling Adequacy		.835
Bartlett's Test of Sphericity	Approx. Chi-Square	4145.567
	Df	45
	Sig.	.000

Total Variance Explained						
Component	Initial Eigenvalues			Extraction Sums of Squared Loadings		
	Total	% of Variance	Cumulative %	Total	% of Variance	Cumulative %
1	3.359	33.593	33.593	3.359	33.593	33.593
2	1.204	12.042	45.635	1.204	12.042	45.635
3	1.077	10.771	56.406	1.077	10.771	56.406
4	.893	8.931	65.337			
5	.740	7.396	72.733			
6	.693	6.926	79.659			
7	.672	6.723	86.382			
8	.518	5.177	91.559			
9	.434	4.345	95.904			
10	.410	4.096	100.000			

Component Matrix ^a^			
	Component		
	1	2	3
Region	-.305	-.490	.569
Setting	.449	.370	-.426
Gender	-.080	.746	.209
Age	-.585	-.128	-.213
Occupation	-.439	.424	.377
Highest Level of Education	.723	-.073	-.078
Infrastructure Access	.710	-.176	-.188
Training & Mentorship	.590	.150	.426
Awareness Level	.754	-.117	.191
Digital Work Engagement Level	.762	.044	.284

Component Score Coefficient Matrix ^a^			
	Component		
	1	2	3
Region	-.091	-.407	.528
Setting	.134	.307	-.396
Gender	-.024	.620	.194
Age	-.174	-.106	-.198
Occupation	-.131	.352	.350
Highest Level of Education	.215	-.061	-.072
Infrastructure Access	.211	-.146	-.176
Training & Mentorship	.176	.124	.396
Awareness Level	.224	-.097	.178
Digital Work Engagement Level	.227	.036	.264

Extraction Method: Principal Component Analysis.

^a^ 3 components extracted.

### Correlation analysis

The study undertook Pearson linear correlation to check how the study variables relate to each other, whose outcomes are presented in Table 5.

**Table 5.** Correlation coefficients.

Correlations											
Variables		Region	Setting	Gender	Age	Occupation	Education Level	Infrastructure Access	Training Mentorship Skill Variable	Overall Awareness Level	Digital Work Engagement Level
Region	Pearson Correlation	1									
	Sig. (2-tailed)										
Setting	Pearson Correlation	-.274 ^**^	1								
	Sig. (2-tailed)	.000									
Gender	Pearson Correlation	-.079 ^**^	.020	1							
	Sig. (2-tailed)	.000	.372								
Age	Pearson Correlation	.104 ^**^	-.180 ^**^	-.063 ^**^	1						
	Sig. (2-tailed)	.000	.000	.005							
Occupation	Pearson Correlation	.096 ^**^	-.113 ^**^	.155 ^**^	.186 ^**^	1					
	Sig. (2-tailed)	.000	.000	.000	.000						
Education Level	Pearson Correlation	-.148 ^**^	.252 ^**^	-.029	-.386 ^**^	-.290 ^**^	1				
	Sig. (2-tailed)	.000	.000	.189	.000	.000					
Infrastructure Access	Pearson Correlation	-.165 ^**^	.258 ^**^	-.104 ^**^	-.279 ^**^	-.313 ^**^	.539 ^**^	1			
	Sig. (2-tailed)	.000	.000	.000	.000	.000	.000				
Training & Mentorship/Skill	Pearson Correlation	-.106 ^**^	.194 ^**^	.009	-.275 ^**^	-.103 ^**^	.274 ^**^	.245 ^**^	1		
	Sig. (2-tailed)	.000	.000	.679	.000	.000	.000	.000			
Awareness Level	Pearson Correlation	-.132 ^**^	.210 ^**^	-.103 ^**^	-.343 ^**^	-.262 ^**^	.427 ^**^	.455 ^**^	.428 ^**^	1	
	Sig. (2-tailed)	.000	.000	.000	.000	.000	.000	.000	.000		
Digital Work Engagement Level	Pearson Correlation	-.150 ^**^	.241 ^**^	-.030	-.383 ^**^	-.190 ^**^	.428 ^**^	.424 ^**^	.491 ^**^	.574 ^**^	1
	Sig. (2-tailed)	.000	.000	.175	.000	.000	.000	.000	.000	.000	
	N	1987	1987	1987	1987	1987	1987	1987	1987	1987	1987

** Correlation is significant at the 0.01 level (2-tailed).

The Pearson correlation coefficient was computed for all study variables to determine the relationships among the factors presented in Table 5. The correlation between digital work engagement and other factors revealed a statistically significant negative relationship between digital work engagement and location (-0.150), age (-0.383), and occupation (-0.190), revealing that the relationship favors those categories rated lower than the mean rating. However, Gender was observed to have a very low and non-significant negative relationship with engagement in digital work, revealing a very weak relationship between these variables, and the possibility that changes in gender participation might not lead to improvement in digital work engagement levels. Other factors showed a moderately significant positive relationship with location setting (0.241), education level (0.428), infrastructure access (0.424), training and mentorship (0.491), and awareness level (0.574), which reveals that changes in these factors lead to positive changes in digital work engagement. Additionally, the correlations among the independent and demographic variables in the study were mostly low and statistically significant (except for relationships with gender variables), revealing no fears of the variables exhibiting autocorrelation problems in the survey model. Kigotho (2025) claims generational gap in the context of has grown making the transition to digital work platforms diverse among generations calling for more intensive support. Women are often overrepresented in part-time and flexible work arrangements across many economies ( International Labour Organization, 2018).

Infrastructure Access, Awareness Level, and Training and Mentorship and Engagement in Digital Work

Skills development programs targeting the growth of engagement in digital work have been observed to dwell in three areas: improvement in infrastructure access, training and mentorship, and awareness level. This study sought to understand the power of these areas of intervention on the level of engagement in digital work, and thus sought to assess the joint relationship of Infrastructure Access, Training & Mentorship, and Awareness Level on Digital Work Engagement, assessed through the linear regression model presented in Table 6.

**Table 6.** Regression model table – Joint effect model.

Model Summary				
Model	R	R Square	Adjusted R Square	Std. Error of the Estimate
1	.656 ^b^	.431	.430	.349890

ANOVA ^a^						
Model		Sum of Squares	df	Mean Square	F	Sig.
1	Regression	183.862	3	61.287	500.620	.000 ^b^
	Residual	242.764	1983	.122		
	Total	426.626	1986			

Coefficients ^a^						
Model		Unstandardized Coefficients		Standardized Coefficients	t	Sig.
		B	Std. Error	Beta		
1	(Constant)	-.074	.014		-5.154	.000
	Infrastructure Access	.359	.037	.187	9.795	.000
	Training & Mentorship	.181	.012	.290	15.425	.000
	Awareness Level	.145	.008	.365	17.859	.000

^a^ Dependent Variable: Digital Work Engagement.

^b^ Predictors: (Constant), Awareness Level, Training & Mentorship, Infrastructure Access.

As presented in Table 6, the model summary shows the correlation (R) and the coefficient of determination (R ^2^). The R value represents a simple correlation and is 0.656, indicating a moderately high degree of correlation. An R ^2^ value of 0.431 indicates that 43.1% of the total variation in the dependent variable, Digital Work Engagement, can be explained by the independent variables of Awareness Level, Training & Mentorship, and Infrastructure Access. In this case, the coefficient of determination is relatively low and explains only a small fraction of the variability in the dependent variable, indicating the presence of other major variables that can explain more than half of the variability in digital work engagement.

The ANOVA table shows how well the regression equation fits the data (i.e., predicts the dependent variable), where the regression model was observed to predict the dependent variable significantly. This is confirmed by the observed p-value (p = 0.000), which is less than 0.05, and indicates that, overall, the regression model statistically significantly predicts the outcome variable and, hence, is a good fit for the data.

The regression model coefficient tables quantified the influence observed earlier by providing the necessary information to predict how engagement in digital work varies from the variations in Awareness Level, Training & Mentorship, Infrastructure Access, as well as by determining whether these factors contribute statistically significantly to the model. All three variables were confirmed to have statistically significant positive relationships with engagement in digital work (Awareness Level: 0.145, Training & Mentorship: 0.181, Infrastructure Access: 0.359), confirming that they play a significant role in enhancing the level of engagement in digital work. This reveals that awareness level, training and mentorship, and infrastructure access have a significant influence on engagement in digital work; hence, interventions to improve these aspects with a view to enhancing digital work participation provide the desired results. The large coefficient for infrastructure access reveals that interventions in this area have a larger multiplier effect on engagement in digital work than training, mentorship, and awareness creation interventions. The World Bank (2026) advocates for a systems-focused approach to digital inclusion calling for investment in digital infrastructure, capacity building and awareness campaigns to create demand and trust in digital work opportunities.

Moderating influence of demographic factors on the relationship between Infrastructure Access and Engagement in Digital Work

**Figure 1.**
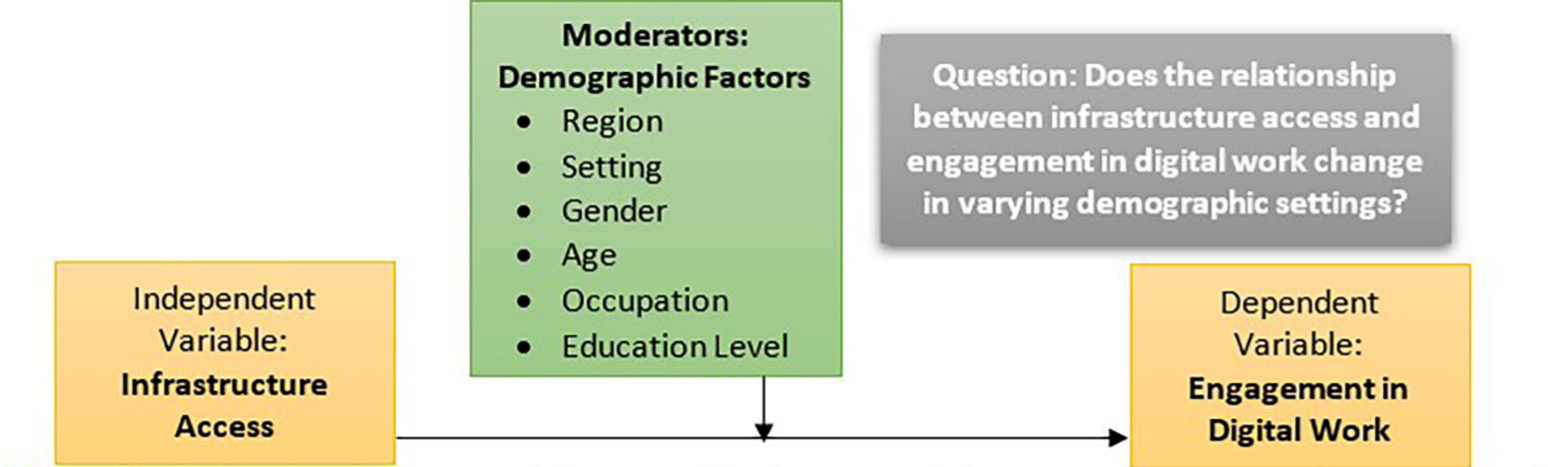
Moderating influence of demographic factors on infrastructure access and engagement in digital work relationship.

This study considered the moderating influence of various demographic factors, including region, setting, gender, age, occupation, and education level, on the relationship between infrastructure access and engagement in digital work. The study adopted a hierarchical regression model, showing all three moderating influence hypotheses. Hierarchical regression is a form of linear regression that allows for comparing different regression models with each model adding 1(+) predictors to the previous model, resulting in a “hierarchy” of models. This approach must satisfy the linear regression assumptions of homoscedasticity, normality, and linearity. This relationship takes the form presented in Figure 1.

To satisfy the moderating effect assessment, the measurement of the causal effect of independent variable X on dependent variable Y for different levels of moderator variable M ensured that the regression model must satisfy the following conditions:

a. **Y = β**_**0**_ **+ β**_**1**_ **X + e** (if statistically significant, there is partial moderation; if not significant, there is full moderation)
b. **Y = β**_**0**_ **+ β**_**1**_ **X + β**_**2**_ **M + β**_**3**_ **XM + e** (relationship between variables, moderator, and interaction (XM) terms). The moderating effects of moderator variable M in the model occur if Hypothesis 3 (β _3_) is significant.

According to Awang (2014), the regression coefficient β _3_ measures the interaction effect between the independent variable X and the moderating variable M. Regression coefficient β1 measures the simple effects of X when the value of M = 0 (no interaction effects involved). The moderation test is then operationalized by the product term XM (the multiplication between the independent variable X and moderator variable M). Centering, subtracting the mean (e.g., x=X-Ẋ) is recommend to reduce nonessential collinearity ( Aiken & West, 1991), which avoids inflation of standard errors that affect the significance tests for the main effects of x and m.

Data analysis revealed the moderating influence of various demographic variables on the relationship between access to infrastructure and engagement in digital work, as presented in **Appendix I: Table x1 (refer to extended data)**. The findings are summarized in Table 7. The variables in the summary table were captured such that Y is Engagement in Digital Work; X is Access to Infrastructure: M1 is Region; M2 is Setting; M3 is Gender; M4 is Age; M5 is occupation, and M6 is educational level.

**Table 7.** Infrastructure access and engagement in digital work demographic moderators (summary table).

Model	R-square change	Statistical significance	Moderating effect
1. Y = 0.037 + 0.814 X + e (Base model)	R ^2^ = 0.180 (base)	X:Sig (p < 0.05)	Direct effect
2. Y = 0.112 + 0.785 X - 0.015M _1_-0.036XM _1_+ e M _1_ Weakens X-Y relationship (small change – 0.8%)	R ^2^ = 0.188; (Change = 0.008)	X:Sig; M _1_:Sig; XM _1_:Sig (p < 0.05)	Low R ^2^ change Partial moderation
3. Y = - 0.083 + 0.748 X + 0.092M _2_ + 0.286XM _2_+ e M _2_ strengthens X-Y relationship (2.6% change)	R ^2^ = 0.206; (Change = 0.026)	X:Sig; M _2_:Sig; XM _2_:Sig (p < 0.05)	Low R ^2^ change Partial moderation
4. Y = 0.813 X + 0.017M _3_ + 0.191XM _3_+ e M _3_ slightly strengthens X-Y relationship (0.2% change)	R ^2^ = 0.182; (Change = 0.002)	X:Sig; M _3_:Non.Sig; XM _3_:Sig (p < 0.05)	Low R ^2^ change Partial moderation
5. Y = 0.324 + 0.655 X - 0.059M _4_-0.154XM _4_+ e M _4_ weakens X-Y relationship (11.1% change)	R ^2^ = 0.291; (Change = 0.111)	X:Sig; M _4_:Sig; XM _4_-Sig (p < 0.05)	Moderate R ^2^ change Partial moderation
6. Y = 0.132 + 0.776 X - 0.021M _5_-0.001XM _5_+ e M _5_ has no moderating effect on X-Y relationship	R ^2^ = 0.183; (Change = 0.003)	X:Sig; M _5_:Sig; XM _5_-No.Sig (p > 0.05)	Low R ^2^ change No moderator effect
7. Y = - 0.258 + 0.491 X + 0.076M _6_ + 0.112XM _6_+ e M6 strengthens X-Y relationship (small change 6.5%)	R ^2^ = 0.245; (Change = 0.065)	X:Sig; M _6_:Sig; XM _6_:Sig (p < 0.05)	Low R ^2^ change Partial moderation

As presented in Table 7, the study found a direct relationship between access to infrastructure and engagement in digital work. Location setting (M _2_), gender (M _3_), and education level (M _6_) strengthened the causal effects of infrastructure access (X) on engagement in digital work (Y), where they were found to be positive partial moderators of this relationship. This is a partial moderation, since the hypothesis for the main effect is statistically significant after the moderator enters the model. Gender was observed to have no direct effect on engagement in digital work, but it was confirmed to have some moderating influence. Further, it was observed that region (M _1_) and age (M _5_) weakened the causal effects of infrastructure access (X) on engagement in digital work (Y), where they were found to be negative partial moderators of this relationship. This means that the causal effect of infrastructure access on engagement in digital work decreases as age increases and increases as it decreases. This confirms that at certain higher age, increase in infrastructure access does not contribute to increase in digital work engagement, hence more digital work programs ought to concentrate in younger people when it comes to interventions related to improving infrastructure access. Given that region was a categorical variable, access to infrastructure translated to digital work engagement among regional categories located lower in the category list, revealing variances in how infrastructure access relates to engagement in digital work in different regions. However, these models show very low moderating effects, lower than 1%. Additionally, although occupation has a significant direct relationship with digital work engagement, the factor was found to have no moderating effect on the relationship with infrastructure access. Therefore, the study confirms that the causal effects of infrastructure access on engagement in digital work are partially moderated by setting (M _2_), gender (M _3_), education level (M _6_), region (M _1_), and age (M _5_), while occupation has no moderating effect. The findings are consistent with a study by Briones, Manaig, Bonganciso, Tesoro, Buama, Sarmiento and Sapin (2023) on the universal applicability of certain capacity-building interventions for digital economy.

Moderating influence of demographics on relationship between training & mentorship and engagement in digital work

**Figure 2.**
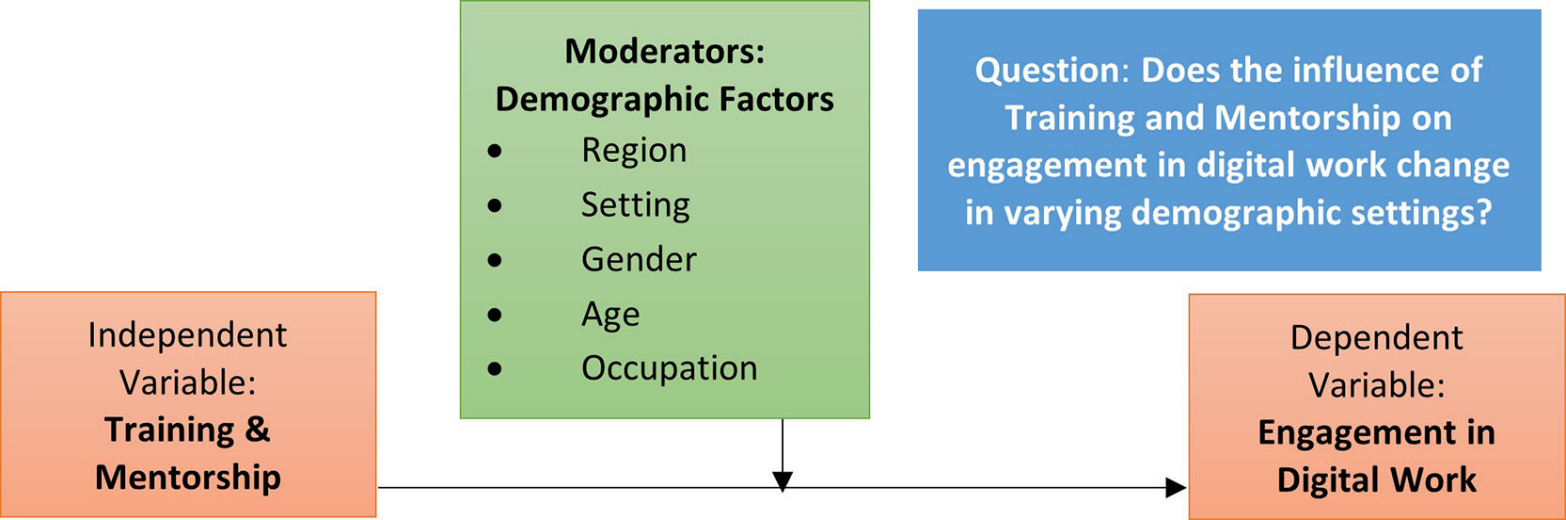
Moderating influence of demographic factors training & Mentorship and engagement in digital work relationship.

This study examined the moderating influence of various demographic factors, including region, setting, gender, age, occupation, and education level, on the relationship between training and mentorship and engagement in digital work. This study adopted a hierarchical regression model to test the three moderating influence hypotheses. The model for this relationship is shown in Figure 2.

The moderating influence of the demographic variables on the relationship between training and mentorship and engagement in digital work was revealed, as presented in **Appendix II: Table x2 (refer to extended data)**. The findings are summarized in Table 8. The variables in the summary table are captured such that Y is Engagement in Digital Work; X is Training & Mentorship: M _1_ is Region; M _2_ is Setting; M _3_ is Gender; M _4_ is Age; M _5_ is occupation, and M _6_ is educational level.

**Table 8.** Training & mentorship and engagement in digital work demographic moderators (summary table).

Model	R-square change	Statistical significance	Moderating effect
1. Y = 0.223 + 0.307 X + e (Base model)	R ^2^ = 0.242 (base)	X:Sig(p < 0.05)	Direct effect
2. Y = 0.313 + 0.308 X - 0.020M _1_ + 0.012XM _1_+ e M _1_ strengthen X-Y relationship (small change 1.2%)	R ^2^ = 0.254; (Change = 0.012)	X:Sig; M _1_:Sig; XM _1_:Sig (p < 0.05)	Low R ^2^ change Partial moderation
3. Y = 0.063 + 0.289 X + 0.116M _2_-0.001XM _2_+ e M _2_ has no moderating effect on X-Y relationship	R ^2^ = 0.264; (Change = 0.022)	X:Sig; M _2_:Sig; XM _2_:No.Sig (p > 0.05)	Low R ^2^ change No moderator effect
4. Y = - 0.145 X + 0.308M _3_ - 0.034XM _3_-0.0.062XM _3 +_ e M _3_ slightly weakens X-Y relationship (0.3% change)	R ^2^ = 0.245; (Change = 0.003)	X:Sig; M _3_:Non.Sig; XM _3_:Sig (p < 0.05)	Low R ^2^ change Partial moderation
5. Y = 0.450 + 0.271 X - 0.049M _4_ + 0.004XM _4_+ e M _4_ has no moderating effect on X-Y relationship	R ^2^ = 0.308; (Change = 0.066)	X:Sig; M _4_:Sig; XM _4_:No.Sig (p > 0.05	Low R ^2^ change No moderator effect
6. Y = 0.411 + 0.301 X - 0.047M _5_ + 0.006XM _5_+ e M _5_ has no moderating effect on X-Y relationship	R ^2^ = 0.261; (Change = 0.019)	X:Sig; M _5_:Sig; XM _5_:No.Sig (p > 0.05	Low R ^2^ change No moderator effect
7. Y = - 0.169 + 0.230 X + 0.083M _6_ + 0.014XM _6_+ e M _6_ has no moderating effect on X-Y relationship	R ^2^ = 0.335; (Change = 0.093)	X:Sig; M _6_:Sig; XM _6_:No.Sig (p > 0.05	Low R ^2^ change No moderator effect

As shown in Table 8, a low significant direct relationship between training and mentorship (X) was confirmed for engagement in digital work (Y), revealing that training and mentorship have a positive influence on engagement in digital work. It was further found that a partial moderator effect, although very low, was confirmed for region (M _1_) and gender (M _3_). The region was found to strengthen the relationship between training and mentorship, and engagement is digital work, revealing that regions in upper categories would enhance engagement in digital work when they receive training and mentorship, unlike those in lower categories. Gender was observed to weaken the causal effects of training and mentorship and engagement in digital work, revealing that the lower gender category – male–has a slightly higher propensity to translate training and mentorship into digital work opportunities.

On the other hand, the study found that setting (M _2_), age (M _4_), occupation (M _5_), and education level (M _6_) had no moderating influence on the relationship between training and mentorship and engagement in digital work. This revealed that these demographic factors had no effect on the causal effect of training and mentorship on training and mentorship. Training and mentorship interventions would spark engagement in digital work, whether in rural, urban, or peri-urban settings, whether among young people or older generations, or well-educated and those with lower educational achievement. This reveals that training and mentorship are very strong tools for improving the level of engagement in digital work, and are not affected by most of the demographic variabilities such as setting, age, occupation, and education level, except for gender and region, which cause very small changes in this relationship. The finding reflects Hayes (2022) assertion that patters are context-specific to digital work environment.

Moderating influence of demographics on relationship between awareness level and engagement in digital work

**Figure 3.**
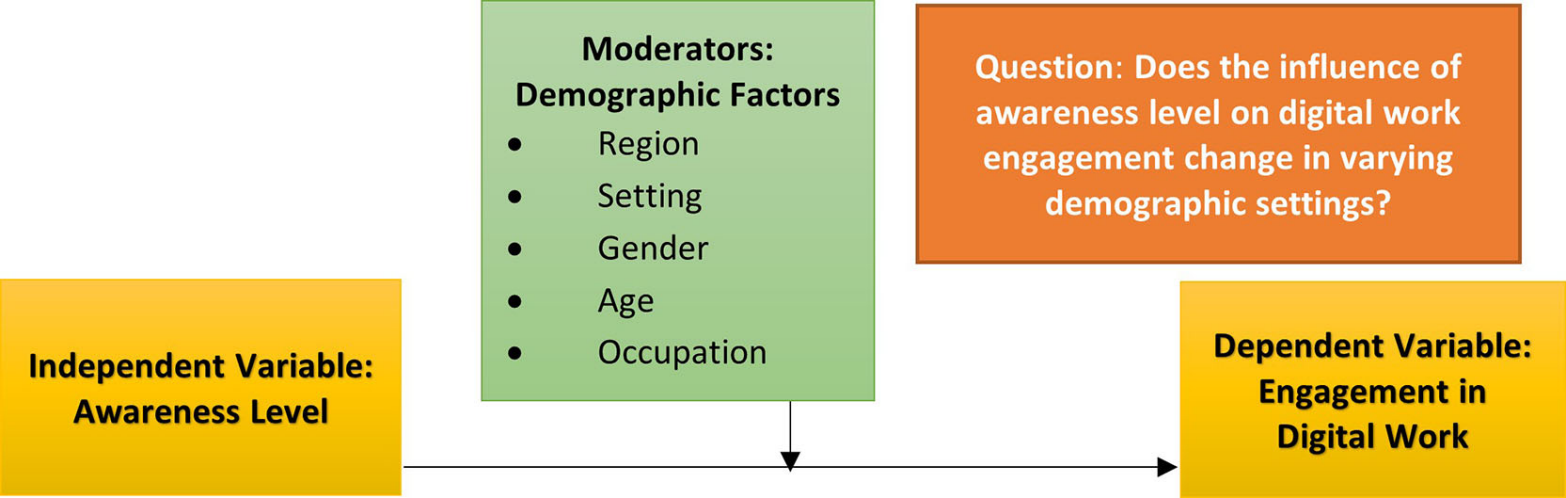
Moderating influence of demographic factors on awareness level and engagement in digital work relationship.

The moderating influence of demographic factors (region, setting, gender, age, occupation, and education level) on the relationship between the awareness level and engagement in digital work was examined. This study adopted a hierarchical regression model to test the three moderating influence hypotheses. The model for this relationship is shown in Figure 3.

The moderating influence of the demographic variables on the relationship between awareness level and engagement in digital work was revealed, as presented in **Appendix III: Table x3 (refer to extended data)**. The findings are summarized in Table 9. The variables in the summary table were captured such that Y is Engagement in Digital Work; X is Awareness Level: M _1_ is Region; M _2_ is Setting; M _3_ is Gender; M _4_ is Age; M _5_ was occupation, and M _6_ was educational level. Li, Fong, Ho, Choi, Lok, Lee, & Lin (2024) in their study on prevalence and factors associated with willingness to sustain Pandemic-Induced digital work in the general population had similar findings.

**Table 9.** Awareness level and engagement in digital work demographic moderators (summary table).

Model	R-square change	Statistical significance	Moderating effect
1. Y = - 0.042 + 0.228 X + e (Base model)	R ^2^ = 0.329 (base)	X:Sig(p < 0.05)	Direct effect
2. Y = 0.223 X - 0.014M _1_-0.009XM _1_+ e M _1_ weakens X-Y relationship (small change 0.9%)	R ^2^ = 0.338; (Change = 0.009)	X:Sig; M _1_:Sig; XM _1_:Sig (p < 0.05)	Low R ^2^ change Partial moderation
3. Y = - 0.145 + 0.213 X - 0.014M _2_-0.009XM _2_+ e M _2_ weakens X-Y relationship (small change 3.2%)	R ^2^ = 0.361; (Change = 0.032)	X:Sig; M _6_:Sig; XM _6_:Sig (p < 0.05)	Low R ^2^ change Partial moderation
4. Y = - 0.086 X + 0.032M _3_ + 0.036XM _3 +_ e M _3_ slightly strengthens X-Y relationship (0.3% change)	R ^2^ = 0.332; (Change = 0.003)	X:Sig; M _3_:Non.Sig; XM _3_:Sig (p < 0.05)	Low R ^2^ change Partial moderation
5. Y = 0.228 + 0.172 X - 0.052M _4_-0.041XM _4_+ e M _4_ weakens X-Y relationship (11.1% change)	R ^2^ = 0.426; (Change = 0.097)	X:Sig; M _4_:Sig; XM _4_:Sig (p < 0.05)	Moderate R ^2^ change Partial moderation
6. Y = 0.222 X - 0.13M _5_-0.021XM _5_+ e M _4_ weakens X-Y relationship (0.2% change)	R ^2^ = 0.331; (Change = 0.002)	X:Sig; M _6_:Sig; XM _6_:Sig (p < 0.05))	Moderate R ^2^ change No moderator effect
7. Y = - 0.324 + 0.167 X + 0.067M _6_ + 0.049XM _6_+ e M6 strengthens X-Y relationship (small change 6.5%)	R ^2^ = 0.411; (Change = 0.082)	X:Sig; M _6_:Sig; XM _6_:Sig (p < 0.05)	Low R ^2^ change Partial moderation

As shown in Table 9, the study found that awareness level has a direct effect on engagement in digital work. This relationship was weakened by regions where the causal effect of awareness level weakens in regions that are located higher within the eight categories of regions highlighted in this study (such as Nyanza, Western, Rift Valley, and Coast), but by a very small margin. Similarly, setting weakens the causal effect of awareness level, with the causal effect being weaker in peri-urban regions or urban and rural settings, which might be linked to the presence of high awareness levels of digital work opportunities in peri-urban and urban settings than in rural settings. Age and occupation also weakened the moderating effect on digital work engagement. This revealed that undertaking awareness drives are more effective among younger people than among older people to spark engagement in digital work. Occupation had a very small effect on the causal effect of awareness level but revealed that occupations listed lower in the occupation categories had higher responsiveness to awareness level to spur engagement in digital work.

However, gender and education level were found to strengthen the causal effect of awareness level on digital work engagement. It was observed that providing awareness to females would have a slightly higher chance of improving their engagement in digital work compared to their male counterparts. Similarly, those with higher education levels have higher responsiveness to awareness creation drives, leading to engagement in digital work, than those with lower education levels. These outcomes reveal that awareness level influence on digital work engagement improves in higher education levels and among the female gender.

## Conclusion and recommendations

Infrastructure access, training and mentorship, and awareness level were directly correlated with digital work engagement level, with demographic factors such as region, gender, age, and occupation having a negative correlation while setting, and education level having positive correlation levels.

Infrastructure access, training, mentorship, and awareness level have a positive influence on the level of engagement in digital work, revealing that improvement in these factors leads to improvement in the level of engagement in digital work. This validates various programs utilizing these three approaches as a means of improving access to digital work in Kenya.

The causal effects of infrastructure access on digital work engagement are strengthened by setting, gender, and education level, which are weakened by region and age, whereas occupation has no moderating effect on this relationship.

The causal effect of training and mentorship on digital work engagement is strengthened by region and weakened by gender, while other factors such as setting, age, occupation, and education level have no moderating effect on this relationship. Therefore, training and mentorship were observed to be strong tools for improving the level of engagement in digital work.

The influence of awareness level on the level of engagement in digital work was found to be weakened by region, setting, age, and occupation, while this relationship was strengthened by gender and education level. This revealed that the causal effect of awareness level on the level of engagement in digital work varies, albeit slightly, in different demographic settings.

## Consent statement

The consent form participants was sufficiently sought.

The study approvals were sought from NACOSTI in Kenya.

## Data Availability

The research data is available online and datasets can be accessed online. DOI: https://doi.org/10.5281/zenodo.18375032 ( Kiambati, K., et al., 2026) Data are available under the terms of the Creative Commons Attribution Non Commercial No Derivatives 1.0 Generic Extended data is available online as Appendix I: Table x1, Appendix II: Table x2, Appendix III: Table x3 https://doi.org/10.5281/zenodo.18548744 (Kiambati, K., et al., 2026) Data are available under the terms of the Creative Commons Attribution Non Commercial No Derivatives 1.0 Generic
