# Supplementary material for: Addressing supply side constraints for digital work creation: A Concurrent Embedded Mixed Method Study in Kenya
Source: Open Res Afr. 2026 Jun 12;9:11. Originally published 2026 Mar 26. [Version 2] doi: 10.12688/openresafrica.16307.2 (PMC13282603; doi:10.12688/openresafrica.16307.2)
Supplement: Peer review history

# Reviewer report: Reviewer response for version 2

**DOI:** 10.21956/openresafrica.17883.r34917

**Published:** 2026-06-19

**Authors:** Dr.B. R. Kumar Kumar, Dr.Ananthaneni Madhuri

**Affiliations:** 1 Andhra Loyola College, Vijayawada, Andhra Pradesh, India; 2 MBA, Andhra Loyola College, Vijayawada, Andhra Pradesh, India

**Dear Editor and Authors,**

 Thank you for providing the revised version of the manuscript, *" Addressing supply side constraints for digital work creation: A Concurrent Embedded Mixed Method Study in Kenya"*.

 The authors have comprehensively addressed the previous feedback. The theoretical framing has been significantly strengthened by explicitly anchoring the study within digital divide theory, human capital theory, and labor market segmentation theory. Furthermore, the operationalization of variables and the integration of the concurrent embedded mixed-methods design have been sufficiently clarified.

 I have no further comments to make on this revised article and believe it is now ready for indexing.

 Sincerely,

Is the work clearly and accurately presented and does it cite the current literature?

Partly

If applicable, is the statistical analysis and its interpretation appropriate?

Partly

Are all the source data underlying the results available to ensure full reproducibility?

No

Is the study design appropriate and is the work technically sound?

Yes

Are the conclusions drawn adequately supported by the results?

Yes

Are sufficient details of methods and analysis provided to allow replication by others?

Partly

Reviewer Expertise:

Marketing, HR, Entrepreneurship, Financial Markets, Digital Marketing, Sales & distribution, Business strategy & Innovation.

We confirm that we have read this submission and believe that we have an appropriate level of expertise to confirm that it is of an acceptable scientific standard.

# Reviewer report: Reviewer response for version 1

**DOI:** 10.21956/openresafrica.17485.r34226

**Published:** 2026-05-18

**Authors:** Dr.B. R. Kumar Kumar, Dr.Ananthaneni Madhuri

**Affiliations:** 1 Andhra Loyola College, Vijayawada, Andhra Pradesh, India; 2 MBA, Andhra Loyola College, Vijayawada, Andhra Pradesh, India

**Summary of the Article and Its Contribution**

 The manuscript examines supply-side constraints influencing engagement in digital work in Kenya, with particular emphasis on infrastructure access, training and mentorship, and awareness creation, alongside the moderating role of demographic factors. Drawing on a concurrent mixed-methods design that integrates a large-scale survey with qualitative insights, the study finds that infrastructure access and awareness are significant determinants of participation in digital work, while demographic variables such as region, age, and education shape patterns of access and engagement.

 The topic is both timely and policy-relevant, especially in the context of persistent youth unemployment and the expanding digital economy in developing countries. The manuscript has the potential to contribute meaningfully to ongoing discussions on digital inclusion and labour market transformation.

**Major Comments**

**1. Theoretical Framing and Literature Integration**

 While the manuscript engages with a broad range of recent literature, the review remains largely descriptive. The theoretical positioning of the study would benefit from greater clarity and depth, particularly in articulating how the present work advances or challenges existing frameworks.

**Recommendation:**

 The authors are encouraged to more explicitly anchor the study within established theoretical perspectives (e.g., digital divide, human capital, or labor market segmentation theories) and to synthesize prior research in a way that highlights the manuscript’s distinct contribution.

**2. Methodological Transparency and Replicability**

 The mixed-methods design is appropriate and constitutes a notable strength of the study. However, certain aspects of the methodology require further elaboration to ensure transparency and replicability.

 Specifically:

- The operationalization of key variables, particularly the construction of ratio scales, is not sufficiently detailed.
- The survey instrument and item structure are not presented.
- The sampling strategy, while described as nationally representative, would benefit from additional clarification.

**Recommendation:**

 Providing detailed information on measurement, sampling procedures, and, where possible, including the survey instrument (e.g., as an appendix) would substantially strengthen the methodological rigor.

**3. Statistical Reporting and Analytical Depth**

 The manuscript employs appropriate analytical techniques, including regression analysis, correlation, and principal component analysis. However, the presentation of statistical results is somewhat limited.

- Key outputs (e.g., coefficients, significance levels, effect sizes) are not fully reported.
- The interpretation of findings remains largely descriptive, with limited engagement with underlying mechanisms or theoretical implications.

**Recommendation:**

 The authors should provide comprehensive statistical reporting and extend the discussion to offer deeper analytical insight, particularly in relation to findings that diverge from expectations.

**4. Discussion and Contribution**

 The discussion section effectively summarizes the findings but would benefit from a more critical and interpretive approach. Greater engagement with broader theoretical and empirical debates would enhance the manuscript’s contribution.

**Recommendation:**

 The authors may consider elaborating on the implications of their findings, particularly in relation to policy design and the broader literature on digital labour markets and inclusion.

**Minor Comments**

- The manuscript would benefit from careful language editing to improve clarity and fluency.
- Some sections, particularly within the methodology, contain minor repetition and could be streamlined.
- Tables are informative but could be more closely integrated with the narrative discussion.
- Certain findings (e.g., relatively low training outcomes) would benefit from additional clarification.

**Strengths of the Manuscript**

- Addresses a highly relevant and policy-significant research problem
- Employs a robust mixed-methods approach
- Utilizes a large and diverse sample, enhancing empirical credibility
- Incorporates demographic heterogeneity, adding analytical nuance
- Offers practical implications for policymakers and development stakeholders

**Limitations**

- Limited theoretical integration
- Insufficient methodological detail for full replication
- Incomplete statistical reporting
- Absence of data transparency
- Discussion could be more analytically developed

**Recommendation**

**Status: Approved with Reservations**

 The manuscript presents a valuable and policy-relevant contribution, supported by a sound research design and a substantial dataset. However, revisions are required to enhance theoretical clarity, methodological transparency, and analytical depth. Addressing these points would significantly strengthen the manuscript and its contribution to the field.

**Final Remarks to the Authors**

 This manuscript engages with an important and timely topic and demonstrates considerable potential. With further refinement—particularly in strengthening theoretical positioning, enhancing methodological clarity, and deepening analytical discussion—the study is well-positioned to make a meaningful contribution to the literature on digital work and inclusive economic development.

Is the work clearly and accurately presented and does it cite the current literature?

Partly

If applicable, is the statistical analysis and its interpretation appropriate?

Partly

Are all the source data underlying the results available to ensure full reproducibility?

No

Is the study design appropriate and is the work technically sound?

Yes

Are the conclusions drawn adequately supported by the results?

Yes

Are sufficient details of methods and analysis provided to allow replication by others?

Partly

Reviewer Expertise:

Marketing, HR, Entrepreneurship, Financial Markets, Digital Marketing, Sales & distribution, Business strategy & Innovation.

We confirm that we have read this submission and believe that we have an appropriate level of expertise to confirm that it is of an acceptable scientific standard, however we have significant reservations, as outlined above.

# Response

**Published:** 2026-05-19

**Authors:** Kellen Kiambati

**Affiliations:** Human Resource Department, Karatina University, Karatina, Nyeri, Kenya

Dear Dr. Kumar and Dr. Madhuri,  I acknowledge receipt of your recommendations, and I am acting on them.  Thank you.

# Reviewer report: Reviewer response for version 1

**DOI:** 10.21956/openresafrica.17485.r34322

**Published:** 2026-04-28

**Authors:** Ayobami Ojebonde

**Affiliations:** 1 University of Ibadan, Ibadan, Nigeria

This article offers a timely intervention in the global discussion on digital entrepreneurship from a supply-side perspective. Unlike most studies, it focuses on supply-side experiences, uncovering the factors and forces that influence access to and participation in the digital economy.

 The study utilizes a mixed-methods approach, combining quantitative and qualitative data to unravel the causes of participation (or lack thereof) in the digital economy. This is a commendable effort. The methodology is sound, the analysis is rigorous, and the nationwide sample effectively covers diverse demographics, including age, gender, location, and infrastructure access.

 However, there are several opportunities to strengthen the paper:

**1. Editorial Quality**

 The paper requires more thorough proofreading. The frequency of language errors significantly discredits the work. For example:

- **Table 1:** "Variables estimates summary descriptive" is unclear.
- **Consistency:** In the list of variables (M1–M6), the text shifts from present tense ("M1 is Region") to past tense ("M5 was occupation"), which disrupts the flow.
- **Clarity:** The phrase "A total of 10 selected based for IDIs..." is incomplete and confusing.

**2. Methodological Rigor**

 Several methodological details require clarification:

- **Design:** The paper (title) claims to be concurrent and embedded. In the methods section, there is need to clarify and explain what makes the paper so. The presentation should also demonstrate the concurrentness and embeddedness.
- **Transparency:** The methodology should state the exact number of in-depth interviews (IDIs) conducted, the selection criteria, or the participant profiles.
- **Analysis:** The method of data analysis for qualitative aspects need more details. And more clarity. Where the authors said "common discourses" were identified, could they actually mean "common themes" (Could it be that their method of analysis was Thematic Analysis?).

**3. Critical Engagement with Findings**

 The findings deserve a more critical discussion, particularly regarding gender:

- **Counterintuitive Results:** The study found that gender has a low, significant negative correlation with participation. This contradicts common trends and is worth highlighting and celebrating. If gender is no longer a primary barrier, what does this mean for policy and activism? Does this suggest that years of gender-focused interventions have finally succeeded? The paper needs a more contextual treatment of such significant findings.

**4. Integration of Qualitative Data**

 The qualitative findings could be much better integrated and interrogated. While ten focus groups and several IDIs were conducted, their data is largely absent from the findings section.

- **Evidence:** The report should include representative quotes to "show" rather than just "tell."
- **Visibility:** Currently, the lack of qualitative evidence makes that portion of the research feel invisible.

In ALL: the paper is a very good paper but there are opportunities to make it even better.

Is the work clearly and accurately presented and does it cite the current literature?

Yes

If applicable, is the statistical analysis and its interpretation appropriate?

Yes

Are all the source data underlying the results available to ensure full reproducibility?

Yes

Is the study design appropriate and is the work technically sound?

Yes

Are the conclusions drawn adequately supported by the results?

Yes

Are sufficient details of methods and analysis provided to allow replication by others?

No

Reviewer Expertise:

Research methods, development studies

I confirm that I have read this submission and believe that I have an appropriate level of expertise to confirm that it is of an acceptable scientific standard.

# Response

**Published:** 2026-05-19

**Authors:** Kellen Kiambati

**Affiliations:** Human Resource Department, Karatina University, Karatina, Nyeri, Kenya

Dear Ayobami,   I acknowledge receipt of your recommendations, and I am acting on them. Thank you.
